# Evaluation of a Sugarcane (*Saccharum* spp.) Hybrid F_1_ Population Phenotypic Diversity and Construction of a Rapid Sucrose Yield Estimation Model for Breeding

**DOI:** 10.3390/plants12030647

**Published:** 2023-02-01

**Authors:** Zhijun Xu, Ran Kong, Dongsheng An, Xuejiao Zhang, Qibiao Li, Huzi Nie, Yang Liu, Junbo Su

**Affiliations:** 1 South Subtropical Crop Research Institute, Chinese Academy of Tropical Agricultural Sciences, Zhanjiang 524091, China; 2 Zhanjiang Experiment Station, Chinese Academy of Tropical Agricultural Sciences, Zhanjiang 524031, China; 3 Guangdong Modern Agriculture (Cultivated Land Conservation and Water-Saving Agriculture) Industrial Technology Research and Development Center, Zhanjiang 524031, China; 4 Zhanjiang Experimental and Observation Station for National Long-Term Agricultural Green Development, Zhanjiang 524031, China; 5 Agro-Tech Extension Center of Guangdong Province, Guangzhou 510520, China; 6 College of Modern Agriculture, Jiaxing Vocational and Technical College, Jiaxing 314036, China

**Keywords:** sugarcane, F_1_ population, phenotypic diversity, sucrose yield estimated model

## Abstract

Sugarcane is the major sugar-producing crop worldwide, and hybrid F_1_ populations are the primary populations used in breeding. Challenged by the sugarcane genome’s complexity and the sucrose yield’s quantitative nature, phenotypic selection is still the most commonly used approach for high-sucrose yield sugarcane breeding. In this study, a hybrid F_1_ population containing 135 hybrids was constructed and evaluated for 11 traits (sucrose yield (SY) and its related traits) in a randomized complete-block design during two consecutive growing seasons. The results revealed that all the traits exhibited distinct variation, with the coefficient of variation (CV) ranging from 0.09 to 0.35, the Shannon-Wiener diversity index (*H*′) ranging between 2.64 and 2.98, and the broad-sense heritability ranging from 0.75 to 0.84. Correlation analysis revealed complex correlations between the traits, with 30 trait pairs being significantly correlated. Eight traits, including stalk number (SN), stalk diameter (SD), internode length (IL), stalk height (SH), stalk weight (SW), Brix (B), sucrose content (SC), and yield (Y), were significantly positively correlated with sucrose yield (SY). Cluster analysis based on the 11 traits divided the 135 F_1_ hybrids into three groups, with 55 hybrids in Group I, 69 hybrids in Group II, and 11 hybrids in Group III. The principal component analysis indicated that the values of the first four major components’ vectors were greater than 1 and the cumulative contribution rate reached 80.93%. Based on the main component values of all samples, 24 F_1_ genotypes had greater values than the high-yielding parent ‘ROC22’ and were selected for the next breeding stage. A rapid sucrose yield estimation equation was established using four easily measured sucrose yield-related traits through multivariable linear stepwise regression. The model was subsequently confirmed using 26 sugarcane cultivars and 24 F_1_ hybrids. This study concludes that the sugarcane F_1_ population holds great genetic diversity in sucrose yield-related traits. The sucrose yield estimation model, $y_{SY}=2.01x_{SN}+8.32x_{SD}+0.79x_{B}+3.44x_{SH}-47.64$, can aid to breed sugarcane varieties with high sucrose yield.

## 1. Introduction

Sugarcane is the most important sugar-producing crop worldwide. It accounts for 85–90% sugar production in China and is mainly distributed in Guangxi, Yunnan, and Guangdong provinces [1,2]. Until 2020, the sugarcane harvest area was about 1.35 × 10^6^ hectares in China, which produced 1.08 × 10^8^ tons of cane with average sugar productivity of 6.99 t/ha [3]. With China’s economic development, the demand for sugar, as a food ingredient and a component of industrial raw materials, is increasing [4]. To meet the increasing demand, as there are limitations by the sugarcane cropping area, the sugarcane per unit sucrose yield needs to be sustainably improved. Therefore, breeding sugarcane cultivars with high sucrose yield performance traits is critical [5].

However, the complex sugarcane genome, the laborious breeding technologies, and large number of phenotypic traits considerably restrict the efficiency of genetic improvement [6,7]. Modern sugarcane cultivars are interspecific hybrids of *Saccharum officinarum* and *S. spontaneum* and may even contain chromosome from other related genera, such as *Erianthus arundinaceus*, *S. robustum*, et al. [8,9,10,11]. Sugarcane cultivar has a complex genome due to variable ploidy levels, with euploid and aneuploid sets of chromosomes (chromosome had 8–12 copies with homologous genes) ranging from 100 to 130, and a large genome size of approximately 10 Gb [12,13,14]. Because of its huge genome size and high ploidy level, genetic studies of sugarcane yield have been extremely challenging and its progress limited. Only a few yield-related quantitative trait loci (QTLs) have been discovered to date and are unusable for genetic improvement [5,12,14,15,16,17,18]. 

In the conventional sugarcane breeding program, a sugarcane cultivar is released after the following stages: (i) crossing between elite parental accessions to generate hundreds of thousands of F_1_ progenies; (ii) selection of the individuals with desirable traits from the seedling nursery; (iii) selection of excellent breeding lines; (iv) line evaluation over several harvests in multi-environment trials; (v) a variety of regional tests. The breeding process is expensive and laborious, and 12 years are approximately required to obtain one or a few cultivars [16,19,20,21]. In addition, the population of F_1_ progenies is about 0.6–1 million seedlings each year in China, which further increase the difficulty and the cost of sugarcane breeding.

Systematic phenotypic characterization is still essential in sugarcane genetic improvement [7]. Phenotypic traits are intuitive manifestations of the breeding population and an important indicator of genetic improvement. The diversity of phenotypic traits involves the comprehensive performance of the genetic diversity and environmental effects [22], which includes both stability and variability [23]. The evaluation of breeding population phenotypic traits is important for understanding population variation, trait characters, and the main constituent factors of sucrose yield. In breeding practice, the huge number of seeds produced during hybridization makes it very difficult for breeders to systematically evaluate the F_1_ population and select the desirable individuals [16]. If the genetic characteristics and key factors of important sugarcane traits are comprehensively dissected, key traits, such as yield, can be selected in the seedling stage, helping reduce the difficulty of selecting excellent individuals for quantitative traits. 

In this study, we constructed an F_1_ population and systematically investigated the phenotypes of 11 sucrose yield-related traits in hybrid F_1_ populations derived from the elite parents ‘ROC22’ and ‘Yacheng 9446’ in 2020 to 2021. Diversity, correlation, cluster, and principal component analysis were conducted to evaluate the F_1_ population’s phenotypic traits and understand their genetic control. Multivariable linear stepwise regression analysis was applied to mine key sucrose yield composition traits and establish a rapid sugar yield estimation model to access the breeding process. This study lays the foundation for understanding the genetic characteristics of sucrose yield composition traits and thus aiding the breeding of high-yield sugarcane varieties.

## 2. Results

### 2.1. Construction of Hybrid F_1_ Population

Primer combinations were used to genotype the parental lines and identify the hybrid plants. In this study, 3 SSR markers (sh020061, sh060101, and sh090229) [24] were detected to be differentially amplified between ‘Yacheng 9446’ and ‘ROC22’ from 20 markers. The primer combinations were used for genotyping the 209 seedlings based on the male parent’s specific bands, and 171 real F_1_ hybrids were detected. Finally, after removing 36 seedlings that died of disease, we constructed an F_1_ population with 135 progenies, and this population was used for the genetic diversity analysis.

### 2.2. Phenotype Variation and Diversity of the Hybrid F_1_ Population 

**Figure 1:**
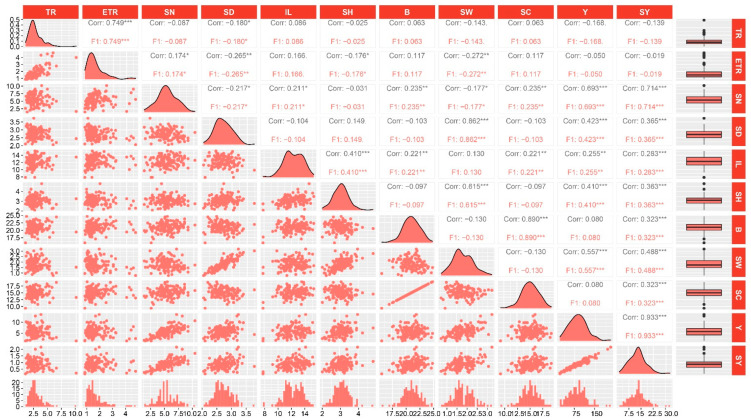
Distribution and correlation analysis of the eleven traits in the hybrid F_1_ population. TR, ETR, SN, SD, IL, SH, B, SW, SC, Y, and SY are the abbreviations of tillering ratio, effective tillering ratio, stalk number, stalk diameter, internode length, stalk height, Brix, stalk weight, sucrose content, yield, and sucrose yield, respectively. For each trait, the frequency distribution (bottom), distribution fitting curve (diagonal), boxplot (right), scatter plot (below diagonal), and correlation coefficient between pairs of traits are shown. *, ** and *** represent significant at *p* < 0.05, *p* < 0.01, and *p* < 0.001, respectively.

The trait name, abbreviations, units, and descriptive statistics of the 11 traits are shown in Table 1. The coefficient of variation (CV) of the 1 traits ranged from 0.09 to 0.35. The tillering ratio (TR), effective tillering ratio (ETR), yield (Y), and sucrose yield (SY) showed a relatively high (greater than 0.30) coefficient of variation, with the highest CV being 0.43 for effective tillering ratio (ETR), indicating that these traits contained rich variation in the hybrid F_1_ population. On the other hand, the traits’ stalk diameter (SD), internode length (IL), Brix (B), and sucrose content (SC) exhibited a relatively small coefficient of variation (CV < 0.15), with Brix (B) having the minimum coefficient of variation (0.09), indicating that these traits were relatively stable in the population. The skewness and the kurtosis ranged from −0.09 to 1.86 and from −0.20 to 5.57, respectively. The Shannon-Wiener diversity index (*H*′) of the traits evaluated ranged between 2.64 and 2.98, indicating abundant phenotypic diversity. The broad-sense heritability of the traits ranged from 0.75 to 0.84. The high heritability indicated that genetic factors played a predominant role in determining trait variation. According to the variance analysis, the environment also significantly influenced the traits (*p* < 0.001). The trait frequency distribution was partial or bimodal, as shown in Figure 1, suggestive of quantitative traits controlled by multiple genes.

### 2.3. Correlation Analysis of the Eleven Traits

Correlation analysis revealed that 30 pairs of the 11 evaluated traits were significantly correlated. Among them, 17 pairs exhibited significant correlation at *p* < 0.001, 7 pairs were significant at *p* < 0.01, and 6 pairs were significant at *p* < 0.05 (Figure 1). Moreover, a positive correlation was observed in 24 pairs of traits, while 6 pairs of traits exhibited a negative correlation. For example, the effective tillering ratio (ETR) exhibited a positive correlation with tillering ratio (TR), while stalk diameter (SD) was negatively correlated with stalk number (SN) but showed a significant positive relationship with stalk weight (SW). Yield (Y) and sucrose yield (SY) exhibited the highest positive correlation coefficient of 0.93, and all were significantly correlated with stalk number (SN), stalk diameter (SD), internode length (IL), stalk height (SH), and stalk weight (SW). Sucrose yield (SY) was also significantly correlated with Brix (B) and sucrose content (SC). Finally, internode length (IL) was significantly correlated with stalk height (SH), Brix (B), sucrose content (SC), yield (Y), and sucrose yield (SY). 

### 2.4. Cluster Analysis 

**Figure 2:**
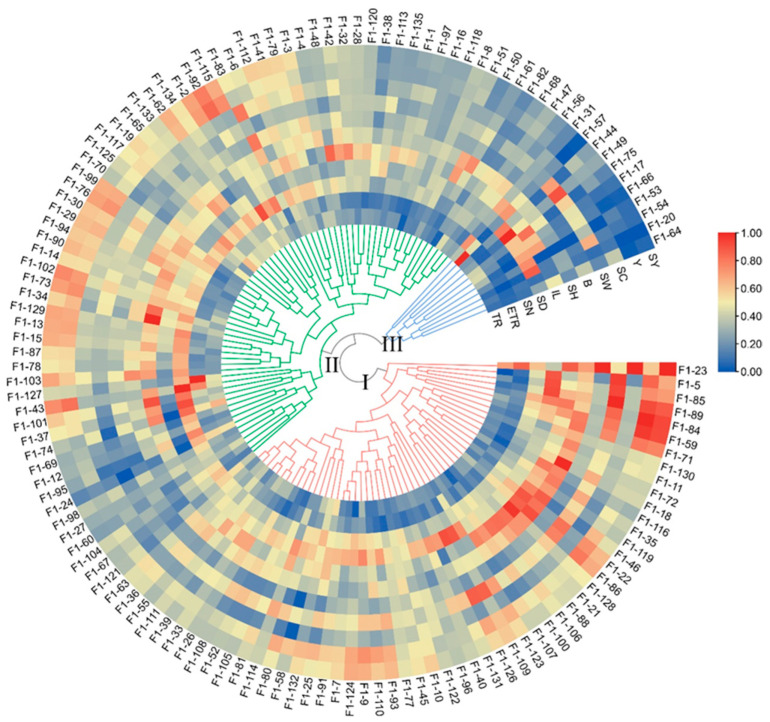
Cluster tree of the sugarcane F_1_ population based on the 11 phenotypic traits. The cluster tree was generated using the standardized traits data through the Ward method. The heatmap of traits was built using the standardized data. TR, ETR, SN, SD, IL, SH, B, SW, SC, Y, and SY are the abbreviations of tillering ratio, effective tillering ratio, stalk number, stalk diameter, internode length, stalk height, Brix, stalk weight, sucrose content, yield, and sucrose yield, respectively. The 135 F_1_ genotypes were divided into 3 groups (Group I-Group III), and the groups are indicated by green, blue, and red lines, respectively.

The Ward method was used for generating a cluster tree of the 11 traits of the 135 F_1_ genotypes. The 135 individuals were clustered into 3 groups with 55 hybrids in Group I, 69 hybrids in Group II, and 11 hybrids in Group III (Figure 2). The main characteristics of Group I were as follows: higher stalk height (SH), wider stalk diameter (SD), longer internode length (IL), greater stalk weight (SW), higher yield (Y), and sucrose yield (SY). Moreover, group two had a higher effective tillering ratio (ETR), more stalk number (SN), and more sugar (brix (B) and sucrose content (SC)). The main characteristics of Group III were higher tillering ratio (TR) but lower effective tillering ratio (ETR) and stalk number (SN), shorter stalk (SH), and relatively low yield (Y) and sucrose yield (SY).

### 2.5. Principal Component Analysis (PCA)

**Figure 3:**
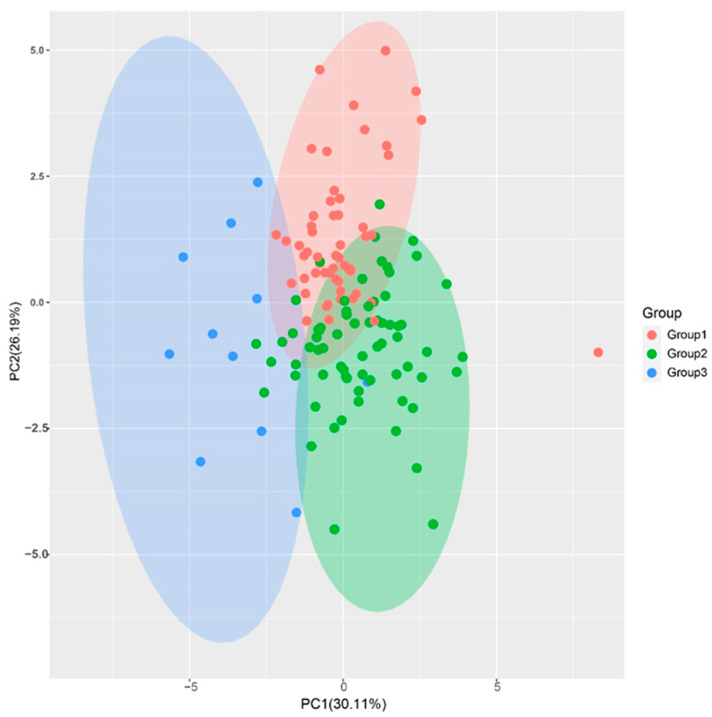
Principal component analysis of the sugarcane hybrid F_1_ population. The plot was drawn using the first two PCs of the F_1_ population. The red, green, and blue dots indicate the F_1_ progenies clustered in Group I, Group II, and Group III, respectively.

PCA is a dimensionality method used to reduce the complexity of large data sets and increase interpretability while minimizing information loss [25]. In this study, the eigenvalues of the first four principal components (PC) were greater than 1, and the cumulative proportion of the first four PCs was 80.93%, indicating that they are representative of most of the 11 traits’ information (Table 2). PC1 had the largest variance proportion, with 30.11%. The trait vectors with the higher values were stalk number (SN), Brix (B), sucrose content (SC), and sucrose yield (SY), indicating that the PC1 was mainly reflecting information about these traits. The PC2 had the second largest variance proportion, with 26.19%. The trait vectors with the higher values were stalk diameter (SD) and stalk weight (SW), indicating that this PC was mainly affected by stalk diameter and weight. The variance proportion of PC3 was 12.97%, and the higher vectors were Brix (B) and sucrose content (SC). Thus, PC3 was mainly affected by traits related to sucrose content. The variance proportion of PC4 was 11.66%, and the higher values vectors were longer internode (IL) and stalk height (SH). The PCA plot of the F_1_ population, drawn using PC1 and PC2, also showed a clear group separation consistent with the cluster tree (Figure 3). The comprehensive evaluation showed that the PC values (F) of all samples were distributed between 12.29 and 46.78, with a median of 29.62. There were 24 F_1_ genotypes with F values greater than the high yield parents ROC22 (35.47), corresponding to 17.8% of the F1 population, with 13 genotypes in Group I and 11 in Group II (Table 3).

### 2.6. Rapid Sucrose Yield Estimation Model Construction and Verification

To establish the mathematical relationship of sucrose yield with its related traits, the sucrose yield (SY) was used as the dependent variable, and its significant correlated traits (*p* < 0.001), stalk height (SH), stalk diameter (SD), stalk number (SN), internode length (IL), stalk weight (SW), Brix (B), stalk weight (SW), and sucrose content (SC), were used as independent variables. Linear regression analysis indicated that sucrose yield (SY) was related to stalk number (SN) (*p* < 0.001), Brix (B) (or sucrose content (SC), *p* < 0.001), and stalk weight (SW) (*p* < 0.05) (Table S1). Subsequently, multivariable linear stepwise regression was performed, which indicated that sucrose yield (SY) was significantly correlated with the three abovementioned traits according to the following equation $y_{SY}=1.97x_{SN}+0.80x_{B}+6.38x_{SW}-34.03$, with an adjusted R^2^ at 0.9328 (Table S2). Considering the convenience of traits directly measured in the field, the equation was rebuilt without stalk weight (SW). Linear regression analysis indicated that stalk height (SH), stalk diameter (SD), stalk number (SN), and Brix (B) were significantly related to sucrose yield (SY) (*p* < 0.001) (Table 4), and multivariable linear stepwise regression had the same result with an adjusted R^2^ of 0.9301 (Table 5). The sucrose yield (SY) regression equation was $y_{SY}=2.01x_{SN}+8.32x_{SD}+0.79x_{B}+3.44x_{SH}-47.64$.

**Figure 4:**
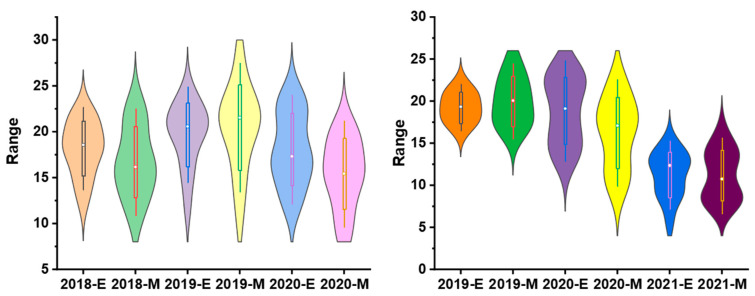
Distribution of the estimated and measured sucrose yield of 14 sugarcane cultivars from 2018 to 2020 and 12 cultivars from 2019 to 2021. 2018-E, 2019-E, 2020-E, and 2021-E represent the estimated sucrose yield, while 2018-M, 2019-M, 2020-M and 2021-M represent the measured sucrose yield in the field.

**Figure 5:**
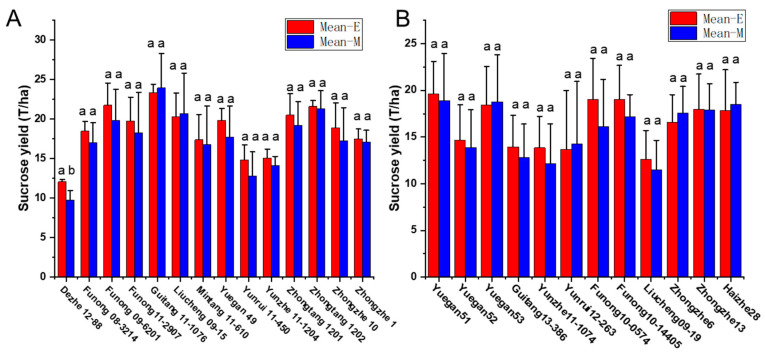
Comparison of the measured and estimated mean sucrose yield of 26 sugarcane cultivars in three crop seasons, including the plant cane, the first ratoon cane, and the second ratoon cane. Mean-E and Mean-M indicate the estimated and measured mean sucrose yield. The sucrose yield of 14 cultivars were measured from 2018 to 2020 in (**A**), and the sucrose yield of the other 12 cultivars were measured from 2019 to 2021 in (**B**). “ab” represent significant differences at *p* < 0.05, and “aa” represent no significant differences at *p* < 0.05.

**Figure 6:**
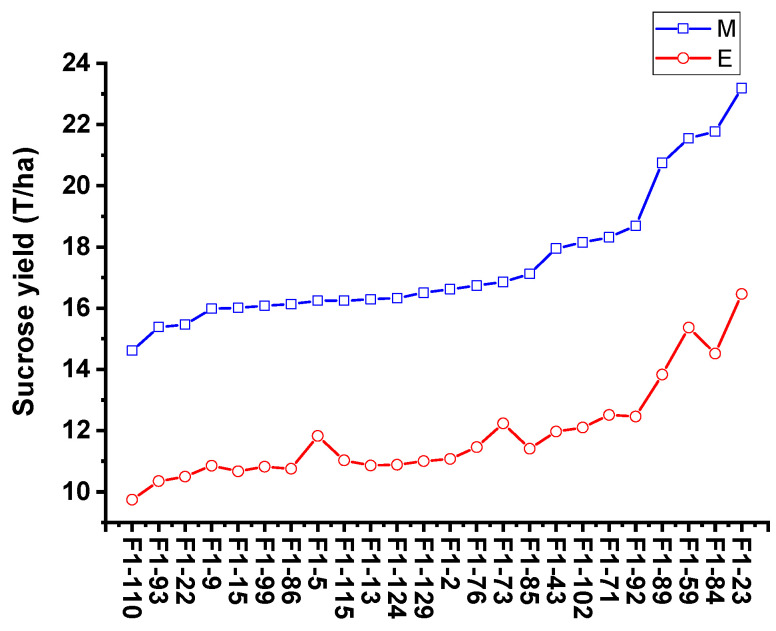
Comparison of the measured mean sucrose yield and estimated sucrose yield distribution trends of 24 F_1_ genotypes. E and M indicate the estimated and measured mean sucrose yield. The estimated sucrose yield of 24 F_1_ genotypes were calculated using the measured stalk height (SH), stalk diameter (SD), stalk number (SN), and Brix (B) of seedling plant.

To validate the equation in genotypes with different genetic backgrounds, the phenotype data from 26 sugarcane cultivars were tested by comparing the estimated and measured sucrose yield. The equation was used to estimate the sucrose yield of the 14 cultivars in 2018–2020 and 12 cultivars in 2019–2021 using the measured data of stalk height (SH), stalk diameter (SD), stalk number (SN), and Brix (B). The estimated sucrose yield exhibited an extremely significant correlation with the measured sucrose yield. The correlation coefficients of the 14 cultivars were 0.9440, 0.9746, and 0.9594 from 2018 to 2020, respectively. Moreover, the correlation coefficients of the 12 cultivars were 0.9806, 0.8772, and 0.8454, respectively. The one-way ANOVA analysis indicated that the estimated and measured sucrose yield variance were not significantly different (*p* < 0.05), and their mean values were not significantly different (*p* < 0.05) (Figure 4, Tables S3 and S4). In addition, the measured mean sucrose yield (SY) of the 26 cultivars in 3 years was compared with the estimated mean sucrose yield (SY). The mean yields were consistent except for the cultivar Dezhe 12–88 (Figure 5). The mean yields of Dezhe 12–88 were significantly different (*p* < 0.05), which may be due to a lot of outliers being recorded for this cultivar that affected the prediction. Further, the sucrose yield (Y) of the 24 F_1_ genotypes (with greater F values than the parents ‘ROC22’) were estimated using the seedling data, and the estimated sucrose yield distribution trends showed a good consistency with the measured mean yield of 2020 and 2021, with correlation coefficients of 0.9783 (Figure 6). All the results above indicated that the equation could be used as a rapid sucrose yield estimation and selection model and may provide valuable reference during the sugarcane breeding.

## 3. Discussion

The combination of the genotype and environmental interactions determines the phenotype. The phenotype is an important manifestation of genetic variation and can directly indicate functional gene diversity [22,26]. In sugarcane, many molecular markers based on genetic diversity have been implemented, such as SSRs [27], RAPDs [28], and SNPs [29], and facilitate the determination of the genetic variability degree occurring among conventional progenitor species, commercial cultivars, and exotic germplasm [30,31]. However, no effective linkage relationship has been established between agronomic traits and molecular markers of sugarcane due to the complexity of the sugarcane genome and the laborious phenotypic measurements at the population level [5,16,31,32]. Thus, assessing the phenotypic and genetic variation among sugarcane germplasm collections or hybrids is still indispensable in providing important information for sugarcane breeding. 

In this study, 11 traits in 135 F_1_ hybrids were evaluated and statistically analyzed. The Shannon–Wiener index of the traits ranged from 2.64 to 2.98, with an average value of 2.89, indicating that the trait diversity was high after the cross of ‘Yacheng 9446’ and ‘ROC22’ and may generate phenotypically overdominant to the parents’ progeny. The coefficient of variation could reflect a trait’s degree of dispersion to a certain extent [33,34]. Usually, when the variation coefficient is greater than 10%, the trait is considered to be diversified among the population [22]. Ten of the eleven traits, except Brix (B), were diversified with a coefficient of variation greater than 10%. Notably, the coefficient of variation of four traits (tillering ratio (TR), effective tillering ratio (ETR), yield (Y), and sucrose yield (SY)) was greater than 30%. Thus, abundant diversity was observed in the traits among the F_1_ population, implying that the F_1_ population parental lines had a good agronomic trait breeding potential. The broad-sense heritability of the 11 traits ranged from 0.75 to 0.84, indicating that they were controlled mainly by genetic factors, consistent with previous research using F_1_ populations and a core sugarcane diversity panel of WCSRG [5,14,15,18]. For example, the broad-sense heritability of stalk number (SN), stalk diameter (SD), and internode length (IL) were 0.88, 0.91, and 0.68 in the core sugarcane diversity panel of WCSRG [5]. Moreover, in ‘R570’ self-crossing F_1_ population, the broad-sense heritability of stalk number (SN), stalk diameter (SD), stalk height (SH) and Brix (B) were 0.81, 0.91, 0.83, and 0.81, respectively [14]. However, the heritability of Brix (B), stalk height (SH), stalk number (SN), and yield (Y) in the Brazilian Panel of Sugarcane Genotypes (a mini core collection) was much lower, with 0.57, 0.57, 0.66, and 0.49 [12], which may be affected by the genetic difference as well as field identification. 

Phenotype clustering can directly reflect the genetic background and be effectively used for a preliminary genetic resource assessment [35]. The cluster analysis divided the 135 F_1_ hybrids into three groups: (1) higher stalk (SH), wider diameter (SD), longer internode (IL), higher stalk weight (SW), yield (Y), and sucrose yield (SY); (2) higher effective tillering ratio (ETR), greater stalk number (SN), and higher sugar (B and SC); (3) higher tillering ratio (TR). This analysis can aid the selection of elite individual F_1_ hybrids and intermediate materials to support the sugarcane breeding programs. The principal component analysis is a comprehensive evaluation method used in the phenotypic evaluation and classifications of rice [36], cotton [37], peanut [38], et al. In this study, the cumulative variance explained by the first four principal components was 80.93%, covering a high proportion of the phenotypic variance. The PCA plot was consistent with the cluster tree dividing the F_1_ population into three groups similarly. By calculating the comprehensive PC values (F) of the F_1_ hybrids and their high-yield parent ‘ROC22’, 24 hybrids with greater F values were selected to proceed to the next breeding stage or be used as intermediate materials.

The sugarcane genome complexity and the quantitative nature of sugar- and yield-related traits are challenging for higher breeding-mediated gains for this crop [12,18]. Correlation analysis showed that stalk number (SN), stalk diameter (SD), internode length (IL), stalk height (SH), and stalk weight (SW) were significantly correlated with yield. These traits, along with Brix (B), sucrose content (SC), and yield (Y), were also significantly correlated with sucrose yield (SY). The relationship between yield (Y) and stalk number (SN), stalk height (SH), and stalk weight (SW) has also been reported by Barreto et al. [5] and Yang et al. [14]. In addition, evidence from genetics also showed that both yield and sucrose were governed by many quantitative trait loci (QTL) or genomic regions [14,39]. These studies also revealed the complexity of high-yield sugarcane breeding and high-sucrose yield breeding. 

To accelerate crop genetic improvement and yield prediction, many efforts have been made. In the main food crop (rice, maize, wheat), genomic selection and phenomic slection have been widely used in breeding [40,41], which need high-precision genome, high-throughput phenome acquisition methods, and high-throughput data analysis methods. For sugarcane, cultivated sugarcane is still lacking a complete genome map and phenotypic analysis model for single breeding line [39]. Therefore, it is important to establish a model between sucrose yield and sucrose yield-related traits for promising single hybrid genotype assessment at the seedling stage. Using the multivariate linear stepwise regression analysis, Liu et al. constructed a yield prediction model for sugarcane in Yunnan province based on plant conditions and meteorological conditions with the accuracy rates of 81.1%, 89.3%, 67.8%, 85.3%, and 73.7%, respectively [42]. Wang et al. constructed a yield model using the grain weight of single sunflower plate, hundred grain weight, seed setting rate, plant height, grain width, and the disc diameter as the main variables, and the model could explain 86.9% of the yield. These studies revealed that multivariate linear stepwise regression is an effective method to construct models [43]. In this study, the significantly correlated traits of sucrose yield were selected to construct the regression equation, resulting in a high adjusted R^2^ of 0.9301. The Brix (B) was selected in the model rather than the sucrose content (SC) due to the convenience of phenotyping. Then, the equation was evaluated by comparing the estimated and measured sucrose yield of 26 new sugarcane cultivars with different genetic background and suitable for different ecological regions in China and sucrose yield distribution trends of 24 F_1_ genotypes. The model could estimate the sugar yield using the stalk height (SH), stalk diameter (SD), stalk number (SN), and Brix (B) values, and could be used at the seedling stage and could be further verified in other stages of sugarcane breeding. In brief, the model can provide a valuable reference for reducing sugar yield evaluation difficulty, aiding for the selection of elite F_1_ lines and reducing time and cost constraints during sugarcane breeding. 

## 4. Materials and Methods

### 4.1. Plant Material 

The F_1_ population was derived from two elite sugarcane resources, Yacheng 9446 and Roc22, which were planted and crossbred at the Hainan sugarcane breeding station (located at Yacheng, Sanya, Hainan, China) in the year 2018. In 2019, the F_1_ seeds were grown into seedlings and planted with the row space of 1.1 m and plant space of 0.33 m in the experimental field of the South subtropical crops research institute (Located at 110°28′54″ W and 21°16′27″ N, Mazhang, Zhanjiang, Guangdong, China). The heterozygosity in hybrid F_1_ plants was confirmed using three differentially amplified SSR markers in the parents we developed previously (Table S5) [24]. DNA of the F_1_ plants were extracted from the young leaves using CTAB method [27]. PCR reactions were conducted following the procedures of Parthiban et al. [27]. The hybrids were identified according to the male parent specific amplified band; once each of the primers could detect the male parent specific band from a F_1_ plant, the plant was a real hybrid. Moreover, if all the three markers could not detect the male parent specific band of a F_1_ plant, the plant was judged to be a false hybrid. Then, the hybrid F_1_ population was planted in the field using two-bud stalk segments and used for phenotyping in 2020 and 2021, respectively. The 26 sugarcane varieties were used as verified materials and their information of all was listed in Table S6.

### 4.2. Field Experiment Design

The experimental design was a randomized complete-block design with three replicates. Each F_1_ hybrid was grown in three rows with a row length of 5.0 m and row space of 1.1 m in each replicate. Twenty-five two-bud stalk segments were planted per row in 2020 and 2021. Sugarcane cultivation management followed that practiced in conventional commercial production in the region, as described by Tang et al. [44]. The 26 sugarcane varieties were planted in the experimental field, adopting the same field experiment design, of which 14 cultivars were planted from the year 2018 to 2020, with the first year newly planted and the two following years stubble sugarcane, and the other twelve were planted from the year 2019 to 2021. All the sugarcane materials were planted in January and harvested in December. 

### 4.3. Phenotypic Traits Identification

Eleven traits, including tillering ratio (TR), effective tillering ratio (ETR), stalk number (SN), stalk diameter (SD), internode length (IL), stalk height (SH), Brix (B), stalk weight (SW), sucrose content (SC), sugarcane yield (Y), and sucrose yield (SY) of the F_1_ population, were measured according to the instructions for sugarcane germplasm resources by Cai et al. [45]. In brief, the tillering ratio (TR) was the ratio of tillering number and main bud seedling number, and effective tillering ratio (ETR) was the ratio of tillering stalk number and tillering number. Main bud seedling number, tillering number, and tillering stalk number of each row were counted per row during sugarcane seedling stage, tillering stage, and harvest stage, respectively. Main bud seedling number and tillering number were counted 10 days a cycle until there were no new gains. Stalk number (SN), stalk diameter (SD), internode length (IL), stalk height (SH), stalk weight (SW), and Brix (B) were measured from 10 random stalks per row 1 week before harvest in December of 2020 and 2021. Stalk diameter (SD) and internode length (IL) were measured at half-height of the stalk using a vernier caliper and meter ruler, respectively. Stalk height was measured using a flexible rule from bottom to the top. Stalk weight (SW) was the mean of 10 stalks weighed per row using an electronic platform scale. Brix (B) was measured using a hand refractometer on the juice taken at half-height of the stalk. Sucrose content (SC) was measured using mixed sample per row determined by HPLC, according to Chen et al. [46]. Yield (Y) was calculated by the stalk number (SN) per unit multiplied by stalk weight (SW). Sucrose yield (SY) was calculated by the Yield (Y) multiplied by the sucrose content (SC). The stalk number (SN), stalk diameter (SD), stalk height (SH), Brix (B), sucrose content (SC), and sucrose yield (SY) of the 26 sugarcane varieties were measured using the same method above.

### 4.4. Phenotypic Diversity and Statistical Analysis

Data statistical analyses. The trait data obtained were sorted using Microsoft Office Excel. The descriptive statistics of each trait, including the overall average ($\bar{x}$), standard deviation (σ), range, variable coefficient, skewness, and kurtosis were calculated using the software Origin 2019b. Data distribution and correlation analysis were analyzed and visualized with the R package ggpairs [47]. The broad-sense heritability was calculated according to Hallauer and Miranda [48] as follows:

h2=σg2σg2+σge2n+σe2nr,

where $\sigma_{g}^{2}$ represents the genetic variance, $\sigma_{ge}^{2}$ represents the interaction variance of the genotype with the environment, $\sigma_{e}^{2}$ represents the variance of residual error, n represents the number of environments, and r represents the number of replications.

Data standardization. The traits data were standardized using the fuzzy membership function method with the following calculation formula:

$$X_{\left(ij\right)}=\frac{X_{ij}-X_{jmin}}{X_{jmax}-X_{jmin}}\;,$$

where $X_{\left(ij\right)}$ represents the membership function value of F_1_ genotype *i* in trait *j*, $X_{ij}$ is the obtained value of the F_1_ genotype *i* in trait *j*, and $X_{jmin}$ and $X_{jmax}$ represent the minimum and maximum values of the F_1_ population in trait j.

Phenotypic diversity. The degree of phenotypic diversity was expressed by the Shannon-Wiener index [49], with the following calculation formula:

$$H^{\prime }=-\overset{n}{\underset{i=1}{\sum }}\left(PilnPi\right)\;\left(i=1,\;2,\;3\ldots \right),$$

where $H^{\prime }$ represents the diversity index, *n* represents the total number of classes, and *Pi* is the effective percentage of the material distribution frequency in the *i-th* class of the trait. The classes were set from the first level < −2σ to the tenth level ≥ 2σ, and every 0.5σ corresponded to one level. 

Cluster analysis. The Ward method was used to generate the cluster tree of 11 traits among 135 F_1_ genotypes, according to the method of [24]. 

Principal component analysis. Principal component analysis (PCA) was carried out using the prcomp function. The 11 traits of the F1 population and the parental line ROC22 were normalized to form a correlation matrix. The correlation matrix was used to determine the eigenvalues and relative contribution rates, and the factor values of the principal components (PC) of each genotype were calculated. All the F_1_ genotypes were comprehensively evaluated according to the eigenvector matrix and phenotypic data. The formulas were as follows:

$$\begin{array}{c} F_{1}=0.19i_{TR}+0.30i_{ETR}+0.40i_{SN}-0.15i_{SD}+0.23i_{IL}+0.15i_{SH}+0.41i_{B}-0.04i_{SW}\\ \;\;\;\;\;\;\;\;\;\;\;\;+0.41i_{SC}+0.33i_{Y}+0.42i_{SY};\\ F_{2}=-0.29i_{TR}-0.27i_{ETR}-0.08i_{SN}+0.46i_{SD}+0.10i_{IL}+0.29i_{SH}-0.07i_{B}+0.54i_{SW}\\ \;\;\;\;\;\;\;\;\;\;\;\;-0.07i_{SC}+0.37i_{Y}+0.30i_{SY};\\ F_{3}=-0.30i_{TR}-0.38i_{ETR}-0.30i_{SN}-0.03i_{SD}-0.01i_{IL}+0.11i_{SH}+0.53i_{B}+0.01i_{SW}\\ \;\;\;\;\;\;\;\;\;\;\;\;+0.53i_{SC}-0.31i_{Y}-0.14i_{SY};\\ F_{4}=-0.23i_{TR}-0.02i_{ETR}+0.19i_{SN}+0.24i_{SD}-0.64i_{IL}-0.58i_{SH}+0.12i_{B}-0.07i_{SW}\\ \;\;\;\;\;\;\;\;\;\;\;\;+0.12i_{SC}+0.17i_{Y}+0.19i_{SY};\;\\ F=0.37F_{1}+0.32F_{2}+0.16F_{3}+0.14F_{4}; \end{array}$$

where *i* represents the F_1_ genotype, and TR, ETR, SN, SD, IL, SH, B, SW, SC, Y, and SY are the trait abbreviations. The 2D-PCA plot was drawn using the R package ggplot [50]. 

### 4.5. Rapid Sugar Yield Estimation Model Construction and Validation

Sugar yield-related traits were selected based on the correlations between the 11 traits. Traits highly significantly correlated with yield or sugar yield (*p* < 0.001) were used as independent variables, and sugar yield was used as the dependent variable. Multivariate linear stepwise regression analysis was performed using the R package MASS to mine the sugar yield composition traits. The sugar yield selection equation was established using the easily quantifiable traits in the field. The sugar yield selection equation was validated using phenotypic data of 26 sugarcane varieties and 24 F_1_ genotypes with greater sucrose yield potential than the high-yield parent ‘ROC22’.

## 5. Conclusions

The sugarcane hybrid F_1_ population, derived from ‘ROC22’ and ‘Yacheng 9446’, exhibited distinct variation and abundant phenotypic diversity. The trait broad-sense heritability and frequency distribution revealed that the 11 traits were controlled by genetic factors and multiple genes. Correlation analysis revealed eight traits significantly associated with the sucrose yield (SY), suggesting the genetic complexity underlying sugarcane high sucrose yield production. Based on the main component values of all samples, 24 F_1_ genotypes had greater values than the high-yield parent ‘ROC22’ and were selected to proceed to the next breeding stage. The rapid sucrose yield estimation equation, $y_{SY}=2.01x_{SN}+8.32x_{SD}+0.79x_{B}+3.44x_{SH}-47.64,$ was established and validated and could be implemented for sucrose yield assessment in sugarcane breeding.

## Figures and Tables

**Table 1:** Characteristics and diversity analysis of sugarcane hybrid F_1_ population.

Traits Name	Abbreviation	Range	Mean ± σ ^a^	CV ^b^	Skewness	Kurtosis	*H*′ ^c^	*h* ^2 d^
Tillering ratio (%)	TR	1.61–10.00	3.89 ± 1.20	0.31	1.40	4.49	2.77	0.81
Effective tillering ratio (%)	ETR	0.94–7.59	2.51 ± 1.07	0.43	1.86	5.06	2.64	0.75
Stalk number (/m2)	SN	1.30–9.30	5.07 ± 1.44	0.28	−0.09	0.61	2.92	0.78
Stalk diameter (Mm)	SD	1.95–3.39	2.61 ± 0.28	0.11	0.40	0.32	2.92	0.80
Internode length (Cm)	IL	9.43–16.78	13.32 ± 1.41	0.11	−0.02	−0.20	2.96	0.84
Stalk height (M)	SH	2.10–4.31	3.16 ± 0.36	0.12	0.30	0.69	2.96	0.83
Brix (%)	B	16.54–30.31	20.87 ± 1.82	0.09	1.16	5.57	2.86	0.80
Stalk weight (Kg)	SW	0.74–2.92	1.72 ± 0.41	0.24	0.53	0.47	2.90	0.82
Sucrose content (%)	SC	10.20–18.71	14.89 ± 1.97	0.13	1.16	5.57	2.86	0.80
Yield (T/ha)	Y	18.75–142.35	77.25 ± 24.00	0.31	0.00	0.16	2.98	0.75
Sucrose yield (T/ha)	SY	2.10–21.75	11.55 ± 4.06	0.35	0.12	0.23	2.98	0.80

^a^ represent standard deviation; ^b^ represent coefficient of variation; ^c^ represent the Shannon-Wiener diversity index; ^d^ represent the broad-sense heritability.

**Table 2:** Variance explained by the first four PCs in the PCA of the F1 hybrid sugarcane population.

Traits	PC1	PC2	PC3	PC4
Tillering ratio	0.19	−0.29	−0.30	−0.23
Effective tillering ratio	0.30	−0.27	−0.38	−0.02
Stalk number	0.40	−0.08	−0.30	0.19
Stalk diameter	−0.15	0.46	−0.03	0.24
Internode length	0.23	0.10	−0.01	−0.64
Stalk height	0.15	0.29	0.11	−0.58
Brix	0.41	−0.07	0.53	0.12
Stalk weight	−0.04	0.54	0.01	−0.07
Sucrose content	0.41	−0.07	0.53	0.12
Yield	0.33	0.37	−0.31	0.17
Sucrose yield	0.42	0.30	−0.14	0.19
Eigenvalue	3.31	2.88	1.43	1.28
Variance proportion (%)	30.11	26.19	12.97	11.66
Cumulative proportion (%)	30.11	56.30	69.27	80.93

PC1, PC2, PC3, and PC4 indicate principal components 1 to 4, respectively.

**Table 3:** Principal component values of 24 F_1_ genotypes.

Genotypes	Group	F_1_	F_2_	F_3_	F_4_	F
F1-2	Group2	65.83	42.62	−20.78	17.76	37.51
F1-5	Group1	66.38	52.41	−25.36	19.44	40.40
F1-9	Group1	64.26	45.21	−20.99	17.21	37.66
F1-13	Group2	63.43	41.99	−17.76	15.66	36.59
F1-15	Group2	62.12	40.15	−15.48	14.43	35.70
F1-22	Group1	63.47	46.51	−20.67	14.06	37.38
F1-23	Group1	72.23	37.50	−8.52	14.52	39.73
F1-43	Group2	71.76	47.57	−26.58	18.28	40.47
F1-59	Group1	76.43	57.54	−28.87	20.94	45.45
F1-71	Group1	67.94	49.56	−22.29	17.97	40.33
F1-73	Group2	66.58	45.47	−21.40	15.47	38.29
F1-76	Group2	64.62	45.50	−19.48	15.96	37.94
F1-84	Group1	78.27	59.97	−30.78	22.12	46.78
F1-85	Group1	64.90	47.41	−19.41	17.18	38.85
F1-86	Group1	66.94	49.02	−24.39	15.17	39.04
F1-89	Group1	73.61	56.37	−26.28	20.84	44.42
F1-92	Group2	70.40	49.96	−25.74	19.13	40.99
F1-93	Group1	59.67	42.24	−16.60	16.10	35.53
F1-99	Group2	62.33	42.47	−17.56	15.02	36.28
F1-102	Group2	66.11	44.26	−18.27	16.21	38.33
F1-110	Group1	60.72	43.78	−18.93	15.66	35.98
F1-115	Group2	65.73	45.10	−22.61	17.18	37.90
F1-124	Group1	62.45	42.94	−16.88	17.18	36.90
F1-129	Group2	63.64	41.55	−16.95	15.56	36.65
ROC22		60.04	41.40	−14.84	14.63	35.47

F_1_, F_2_, F_3_, and F_4_ indicate principal component score values of PC1, PC2, PC3, and PC4, respectively. F indicates the comprehensive principal component value and is calculated according to the ratio of F_1_, F_2_, F_3_, and F_4_ corresponding to each principal component.

**Table 4:** Linear-regression analysis between sucrose yield and six related traits.

	Coefficient	Standard Error	t Value	*p* (>|t|)
Intercept	−47.86	1.77	−26.97	<2 × 10^−16^ ***
Stalk number	2.01	0.07	28.55	<2 × 10^−16^ ***
Stalk diameter	8.34	0.35	23.62	<2 × 10^−16^ ***
Internode length	0.03	0.08	0.39	0.69
Stalk height	3.38	0.29	11.86	<2 × 10^−16^ ***
Brix	0.79	0.05	14.48	<2 × 10^−16^ ***
Sucrose content	NA	NA	NA	NA

*** indicates *p* <0.001.

**Table 5:** Multivariate linear stepwise regression between sucrose yield and related traits.

	Coefficient	Standard Error	t Value	*p* (>|t|)
Intercept	−47.64	1.68	−28.39	<2 × 10^−16^ ***
Stalk number	2.01	0.07	28.86	<2 × 10^−16^ ***
Stalk diameter	8.32	0.35	23.77	<2 × 10^−16^ ***
Stalk height	3.44	0.25	13.55	<2 × 10^−16^ ***
Brix	0.79	0.05	14.59	<2 × 10^−16^ ***

*** indicates *p* <0.001.

## Data Availability

The phenotypic data is available on request from the corresponding author.

## Supplementary Materials

The following supporting information can be downloaded at: https://www.mdpi.com/article/10.3390/plants12030647/s1, Table S1: Linear-regression analysis between sucrose yield and seven related traits; Table S2: Multivariate linear stepwise regression between sucrose yield and three related traits; Table S3: one-way ANOVA analysis of the estimated and measured sucrose yield of the 14 cultivars from 2018 to 2020; Table S4: one-way ANOVA analysis of the estimated and measured sucrose yield of the 12 cultivars from 2019 to 2021; Table S5: differential amplification SSR marker information; Table S6 Information of cultivars used as verified materials.

## References

[1] Que Y., Pan Y., Lu Y., Yang C., Yang Y., Huang N., Xu L. (2014). Genetic analysis of diversity within a Chinese local sugarcane germplasm based on start codon targeted polymorphism. BioMed Res. Int..

[2] Zhang B.Q., Huang Y.X., Zhou Z.F., Zhou S., Duan W.X., Yang C.F., Gao Y.J., Zhang G.M., Song X.P., Zhang X.Q. (2022). Cold-Induced physiological and biochemical alternations and proteomic insight into the response of *Saccharum spontaneum* to low temperature. Int. J. Mol. Sci..

[3] National Bureau of Statistics. https://data.stats.gov.cn/.

[4] Zhang M., Govindaraju M., De Oliveira A. (2018). Sugarcane production in China. Sugarcane—Technology and Research.

[5] Yang X., Luo Z., Todd J., Sood S., Wang J. (2020). Genome-wide association study of multiple yield traits in a diversity panel of polyploid sugarcane (*Saccharum* spp.). Plant Genome..

[6] Filho J.A.D., Calsa Júnior T., Simões Neto D.E., Souto L.S., Souza A.D.S., de Luna R.G., Gomes-Silva F., Moreira G.R., Cunha-Filho M., Pinto Dos Santos A.L. (2021). Genetic divergence for adaptability and stability in sugarcane: Proposal for a more accurate evaluation. PLoS ONE.

[7] Voss-Fels K.P., Wei X., Ross E.M., Frisch M., Aitken K.S., Cooper M., Hayes B.J. (2021). Strategies and considerations for implementing genomic selection to improve traits with additive and non-additive genetic architectures in sugarcane breeding. Theor. Appl. Genet..

[8] Al-Janabi S.M., Honeycutt R.J., McClelland M., Sobral B.W. (1993). A genetic linkage map of *Saccharum spontaneum* L. ‘SES 208’. Genetics.

[9] Silva J.A., Sorrells M.E., Burnquist W.L., Tanksley S.D. (1993). RFLP linkage map and genome analysis of *Saccharum spontaneum*. Genome.

[10] Zhang J., Zhang Q., Li L., Tang H., Zhang Q., Chen Y., Arrow J., Zhang X., Wang A., Miao C. (2019). Recent polyploidization events in three Saccharum founding species. Plant Biotechnol. J..

[11] Garsmeur O., Droc G., Antonise R., Grimwood J., Potier B., Aitken K., Jenkins J., Martin G., Charron C., Hervouet C. (2019). A mosaic monoploid reference sequence for the highly complex genome of sugarcane. Nat. Commun..

[12] Barreto F.Z., Rosa J.R.B.F., Balsalobre T.W.A., Pastina M.M., Silva R.R., Hoffmann H.P., de Souza A.P., Garcia A.A.F., Carneiro M.S. (2019). A genome-wide association study identified loci for yield component traits in sugarcane (*Saccharum* spp.). PLoS ONE.

[13] Vieira M.L.C., Almeida C.B., Oliveira C.A., Tacuatiá L.O., Munhoz C.F., Cauz-Santos L.A., Pinto L.R., Monteiro-Vitorello C.B., Xavier M.A., Forni-Martins E.R. (2018). Revisiting meiosis in sugarcane: Chromosomal irregularities and the prevalence of bivalent configurations. Front. Genet..

[14] Hoarau J.Y., Grivet L., Offmann B., Raboin L.M., Diorflar J.P., Payet J., Hellmann M., D’Hont A., Glaszmann J.C. (2002). Genetic dissection of a modern sugarcane cultivar (*Saccharum spp*.).II. detection of QTLs for yield components. Theor. Appl. Genet..

[15] Ukoskit K., Posudsavang G., Pongsiripat N., Chatwachirawong P., Klomsa-Ard P., Poomipant P., Tragoonrung S. (2019). Detection and validation of EST-SSR markers associated with sugar-related traits in sugarcane using linkage and association mapping. Genomics.

[16] Balsalobre T.W., da Silva Pereira G., Margarido G.R., Gazaffi R., Barreto F.Z., Anoni C.O., Cardoso-Silva C.B., Costa E.A., Mancini M.C., Hoffmann H.P. (2017). GBS-based single dosage markers for linkage and QTL mapping allow gene mining for yield-related traits in sugarcane. BMC Genom..

[17] Racedo J., Gutiérrez L., Perera M.F., Ostengo S., Pardo E.M., Cuenya M.I., Welin B., Castagnaro A.P. (2016). Genome-wide association mapping of quantitative traits in a breeding population of sugarcane. BMC Plant Biol..

[18] Aitken K.S., Hermann S., Karno K., Bonnett G.D., McIntyre L.C., Jackson P.A. (2008). Genetic control of yield related stalk traits in sugarcane. Theor. Appl. Genet..

[19] Dal-Bianco M., Carneiro M.S., Hotta C.T., Chapola R.G., Hoffmann H.P., Garcia A.A., Souza G.M. (2012). Sugarcane improvement: How far can we go?. Curr. Opin. Biotechnol..

[20] Balsalobre T.W.A., Mancini M.C., Pereira G.d.S., Anoni C.O., Barreto F.Z., Hoffmann H.P., de Souza A.P., Garcia A.A.F., Carneiro M.S. (2016). Mixed Modeling of Yield Components and Brown Rust Resistance in Sugarcane Families. Agron. J..

[21] Guilly S., Dumont T., Thong-Chane A., Barau L., Hoarau J.Y. (2017). Analysis of multienvironment trials (MET) in the sugarcane breeding program of Réunion Island. Euphytica.

[22] Xin Y.H., Wu Y.X., Qiao B., Su L., Xie S.Q., Ling P. (2022). Evaluation on the phenotypic diversity of Calamansi (*Citrus microcarpa*) germplasm in Hainan island. Sci. Rep..

[23] Wang Y., Wu G., Zhao A., Li D. (2014). Phenotypic genetic diversity of jujube germplasm resources. Sci. Silvae Sin..

[24] Xu Z., Zhao S., Hu X., Kong R., Su J., Liu Y. (2020). Development, Characterization and speciality of microsatellite markers in AP85-441 and R570 genomic reference sequences. Chin. J. Trop. Crops.

[25] Welch N.G., Madiona R.M., Payten T.B., Jones R.T., Brack N., Muir B.W., Pigram P.J. (2016). Surface adsorbed antibody characterization using ToF-SIMS with principal component analysis and artificial neural networks. Langmuir.

[26] Zhao C., Wang R.Z., Li Y.H., Qiu L.J., Zhao X.W., Guo B.F. (2019). Comprehensive analysis and evaluation of the phenotype and quality traits of Jiangxi soybean germplasm resources. Soybean Sci..

[27] Parthiban S., Govindaraj P., Senthilkumar S. (2018). Comparison of relative efficiency of genomic SSR and EST-SSR markers in estimating genetic diversity in sugarcane. 3 Biotech.

[28] Singh P., Singh S.P., Tiwari A.K., Sharma B.L. (2017). Genetic diversity of sugarcane hybrid cultivars by RAPD markers. 3 Biotech.

[29] Zhang H., Lin P., Liu Y., Huang C., Huang G., Jiang H., Xu L., Zhang M., Deng Z., Zhao X. (2022). Development of SLAF-sequence and multiplex SNaPshot panels for population genetic diversity analysis and construction of DNA Fingerprints for Sugarcane. Genes.

[30] Singh R.B., Mahenderakar M.D., Jugran A.K., Singh R.K., Srivastava R.K. (2020). Assessing genetic diversity and population structure of sugarcane cultivars, progenitor species and genera using microsatellite (SSR) markers. Gene.

[31] Zhang J., Zhang X., Tang H., Zhang Q., Hua X., Ma X., Zhu F., Jones T., Zhu X., Bowers J. (2018). Allele-defined genome of the autopolyploid sugarcane *Saccharum spontaneum* L.. Nat. Genet..

[32] You Q., Yang X., Peng Z., Islam M.S., Sood S., Luo Z., Comstock J., Xu L., Wang J. (2019). Development of an Axiom Sugarcane100K SNP array for genetic map construction and QTL identification. Appl. Genet..

[33] Xu H., Liu M., Dong S., Wu Y., Zhang H. (2019). Diversity and geographical variations of germplasm resources of Armeniaca mandshurica. Chin. J. Plant. Ecol..

[34] Liu J.Q., Yin M.Y., Zuo S.Y., Yang S.B., Wuyun T. (2017). Phenotypic variations in natural populations of Amygdalus pedunculata. Chin. J. Plant. Ecol..

[35] Sun D.L., Bian N.F., Chen Z.D., Xing X.H., Xu Z.J., Qi Y.J., Wang W. (2018). Comprehensive evaluation and index screening of phenotypic traits in peanut germplasm resources. J. Plant. Genet. Resour..

[36] Lin H.B., Wan Y., Xia L.I., Lei J.G., Luo X.D., Yan W.G., Xie J.K. (2012). Analysis on genetic diversity of phenotypic traits in rice (oryza sativa) core collection and its comprehensive assessment. Acta Agron. Sin..

[37] Dai P.H., Sun J.L., He S.P., Wang L.R., Jia Y.H., Pan Z.E., Wang M. (2016). Comprehensive evaluation and genetic diversity analysis of phenotypic traits of core collection in upland cotton. Sci. Agric. Sin..

[38] Xu Z., Wu X., Hu X., Liu Y. (2021). Phenotypic diversity of 33 introduced peanut germplasm accessions and preliminary adaptability evaluation in West Guangdong. Chin. J. Trop. Crops.

[39] Mahadevaiah C., Appunu C., Aitken K., Suresha G.S., Vignesh P., Mahadeva Swamy H.K., Valarmathi R., Hemaprabha G., Alagarasan G., Ram B. (2021). Genomic selection in sugarcane: Current status and future prospects. Front. Plant Sci..

[40] Xu S., Zhu D., Zhang Q. (2014). Predicting hybrid performance in rice using genomic best linear unbiased prediction. Proc. Natl. Acad. Sci. USA.

[41] Xiao Y., Jiang S., Cheng Q., Wang X., Yan J., Zhang R., Qiao F., Ma C., Luo J., Li W. (2021). The genetic mechanism of heterosis utilization in maize improvement. Genome Biol..

[42] Wang P., Chen Y., Li W., Liu S., Liu Y., Zhao G. (2016). The effect of the botanical traits on the yield model in broomrape resistant sunflower. Crops.

[43] Liu Z., Zhao J., Hu R., Liu L. (2023). Yield prediction model for sugarcane in yunnan province based on association rules and multiple linear regression. Guangdong Agric. Sci..

[44] Tang X., Meng X., Jiang J., Huang Z., Wu H., Liu J., He L., Xiong F., Zhong R., Han Z. (2020). Effects of sugarcane/peanut intercropping on soil microenvironment in different plough layer. Chin. J. Oil Crop Sci..

[45] Cai Q., Fan Y.H., Ma L., Ying X.M., Huang Y.K., Wang L.P. (2006). Descriptors and Data Standard for Sugarcane (Saccharum officinarum L.).

[46] Chen D., Zhou W., Yang J., Ao J., Huang Y., Shen D., Jiang Y., Huang Z., Shen H. (2021). Effects of Seaweed Extracts on the Growth, Physiological Activity, Cane Yield and Sucrose Content of Sugarcane in China. Front. Plant Sci..

[47] ggpairs. https://ggobi.github.io/ggally/articles/ggpairs.html.

[48] Hallauer A.R., Miranda J.B. (1898). Quantitative Genetics in Maize Breeding.

[49] Miller L., Pisacane C., Vicino G. (2016). Relationship between behavioural diversity and faecal glucocorticoid metabolites: A case study with cheetahs (*Acinonyx Jubatus*). Anim. Welf..

[50] Wickham H. (2006). ggplot: An Implementation of the Grammar of Graphics. R Package Version 04 0. https://pdfs.semanticscholar.org/ba19/d814f1b02664b4621d90a7e8e27c0eb22515.pdf.

