# Supplementary material for: Evaluation of a Sugarcane (Saccharum spp.) Hybrid F1 Population Phenotypic Diversity and Construction of a Rapid Sucrose Yield Estimation Model for Breeding
Source: Plants (Basel). 2023 Feb 1;12(3):647. doi: 10.3390/plants12030647 (PMC9919227; doi:10.3390/plants12030647)
Supplement: Supplementary file 1 [file plants-12-00647-s001.zip › plants-2154817-supplementary.pdf]

Supplementary Materials

Table S1 Linear-regression analysis between sucrose yield and seven related traits

	Coefficient	Standard error	t value	P (>|t|)
Intercept	-34.03	5.88	-5.79	5.19×10^{-8}***
SN	1.99	0.07	28.62	$<2\times10^{-16}$***
SD	3.02	2.19	1.38	0.1698
IL	0.02	0.07	0.24	0.808
SH	1.19	0.93	1.28	0.2047
B	0.80	0.05	14.89	$<2\times10^{-16}$***
SW	4.11	1.67	2.46	0.0152*
SC	NA	NA	NA	NA

Table S2 Multivarite linear stepwise regression between sucrose yield and three related traits

	Coefficient	Standard error	t value	P (>|t|)
Intercept	-26.05842	1.15584	-22.55	$<2\times10^{-16}$***
SN	1.97614	0.06769	29.2	$<2\times10^{-16}$*****
B	0.8002	0.05228	15.3	$<2\times10^{-16}$****
SW	6.38237	0.22508	28.36	$<2\times10^{-16}$***

Table S3 One-way ANOVA analysis of the estimated and measured sucrose yield of the 14 cultivars from 2018 to 2020

Year	ANOVA	DF[a]	SS[b]	MS[c]	F Value	Prob>F
	Model	1	15.27333	15.27333	1.26768	0.27049
2018	Error	26	313.2559	12.0483		
	Total	27	328.5292			
	Model	1	4.46076	4.46076	0.26193	0.61312
2019	Error	26	442.7934	17.03052		
	Total	27	447.2542			
	Model	1	49.78078	49.78078	3.24935	0.08306
2020	Error	26	398.3259	15.32023		
	Total	27	448.1066			

[a] indicate degree of freedom; [b] indicate sum of squares; [c] indicate mean square.

Table S4 One-way ANOVA analysis of the estimated and measured sucrose yield of the 12 cultivars from 2019 to 2021

Year	ANOVA	DF[a]	SS[b]	MS[c]	F Value	Prob>F
	Model	1	3.35428	3.35428	0.54616	0.4677
2019	Error	22	135.1145	6.14157		
	Total	23	138.4687			
	Model	1	40.50769	40.50769	2.40094	0.13553
2020	Error	22	371.1746	16.87157		
	Total	23	411.6822			
2021	Model	1	0.05753	0.05753	0.00701	0.93403

Error	22	180.5233	8.20561	
Total	23	180.5808		

[a] indicate degree of freedom; [b] indicate sum of squares; [c] indicate mean square.

Table S5 Differential amplification SSR marker information

NO.	Marker name	Forward primer	Reverse primer
1	sh020061	TCTCCATGATAAACCGTATGTGA	TGGCCGGTTTTACAAAGTTC
2	sh060101	ACTTCGTTTCCTGTTGTGGC	CCGTTGTTGTTGCTGTTGTT
3	sh090229	AGAAGCCCATCTCCAGGAAG	TAGTCGGCGAGGTACAGAGG

Table S6 Information of cultivars used as verified materials

NO.	Cultivars	Parents	Breeder
1	Dezhe 12-88	ROC22×Yunrui 05-649	Dehong Sugarcane Research Institute
2	Funong 08-3214	Funong 91-4710×Yuetang 99-240	National Engineering Research Center for Sugarcane
3	Funong 09-6201	Yuetang 92-1287×Yuetang00-319	National Engineering Research Center for Sugarcane
4	Funong11-2907	Guitang 96-211×Yunrui 05-679	National Engineering Research Center for Sugarcane
5	Guitang 11-1076	Guitang 01-212×ROC10	Sugarcane Research Institute，Guangxi Academy of Agricultural Sciences
6	Liucheng 09-15	Liucheng 03/1137 × Roc25	Sugarcane Research Center of Liucheng County
7	Mintang 11-610	Yuenong 73-204×CP72-1210	Research Institute of sugarcane, Fujian Academy of Agricultural Sciences
8	Yuegan 49	Yuetang 91-976×ROC23	Guangdong Provincial Bioengineering Institute (Guangzhou Sugarcane Industry Research Institute)
9	Yunrui 11-450	Yunrui 05-649×ROC22	Ruili Breeding Station of Sugarcane Research Institute, Yunnan Academy of Agricultural Sciences
10	Yunzhe 11-1204	Funong 94-0403×Yunrui 05-679	Sugarcane Research Institute, Yunnan Academy of Agricultural Sciences
11	Zhongtang 1201	Reyin 1×ROC22	Institute of Tropical Bioscience and Biotechnology, Chinese Academy of Tropical Agricultural Sciences
12	Zhongtang 1202	Yuetang 99-66×Neijiang 03-218	Institute of Tropical Bioscience and Biotechnology, Chinese Academy of Tropical Agricultural Sciences
13	Zhongzhe 10	Introduced material	Collaborative Innovation Center of Sugarcane Industry, Guangxi University
14	Zhongzhe 1	ROC25×Yunzhe 89-7	Collaborative Innovation Center of Sugarcane Industry, Guangxi University
15	Yuegan 51	ROC22× HoCP00-1142	Guangdong Provincial Bioengineering

			Institute (Guangzhou Sugarcane Industry Research Institute)
16	Yuegan 52	Yuetang 91-976×Yuetang 93-159	Guangdong Provincial Bioengineering Institute (Guangzhou Sugarcane Industry Research Institute)
17	Yuegan 53	Yuetang 94-128×Yuetang 00-319	Guangdong Provincial Bioengineering Institute (Guangzhou Sugarcane Industry Research Institute)
18	Guitang 13-386	Yuetang 91-976×ROC11	Sugarcane Research Institute， Guangxi Academy of Agricultural Sciences
19	Yunzhe 11-11074	Guitang 00-173×ROC22	Sugarcane Research Institute, Yunnan Academy of Agricultural Sciences
20	YunRui 12-263	HoCP95-988×Yunrui 05-770	Ruili Breeding Station of Sugarcane Research Institute, Yunnan Academy of Agricultural Sciences
21	Funong 10-0574	CP72-1210×Yunzhe 94-343	National Engineering Research Center for Sugarcane
22	Funong 10-14405	Yuetang 91-976×Mintang 86-05	National Engineering Research Center for Sugarcane
23	Liucheng 09-19	Liucheng 03/1137 × Roc25	Sugarcane Research Center of Liucheng County
24	Zhongzhe 6	ROC25×Yunzhe 89-7	Collaborative Innovation Center of Sugarcane Industry, Guangxi University
25	Zhongzhe 13	HoCP01-157×CP14-0969	Collaborative Innovation Center of Sugarcane Industry, Guangxi University
26	Haizhe 28	Guitang 94-119×Yacheng 06-63	Hainan Sugarcane Breeding Station of Guangdong Provincial Bioengineering Institute
